# Protein Flexibility Facilitates Quaternary Structure Assembly and Evolution

**DOI:** 10.1371/journal.pbio.1001870

**Published:** 2014-05-27

**Authors:** Joseph A. Marsh, Sarah A. Teichmann

**Affiliations:** 1 European Molecular Biology Laboratory, European Bioinformatics Institute, Wellcome Trust Genome Campus, Hinxton, Cambridge, United Kingdom; 2 Wellcome Trust Sanger Institute, Wellcome Trust Genome Campus, Hinxton, Cambridge, United Kingdom; Brandeis University, United States of America

## Abstract

The intrinsic flexibility of proteins allows them to undergo large conformational fluctuations in solution or upon interaction with other molecules. Proteins also commonly assemble into complexes with diverse quaternary structure arrangements. Here we investigate how the flexibility of individual protein chains influences the assembly and evolution of protein complexes. We find that flexibility appears to be particularly conducive to the formation of heterologous (i.e., asymmetric) intersubunit interfaces. This leads to a strong association between subunit flexibility and homomeric complexes with cyclic and asymmetric quaternary structure topologies. Similarly, we also observe that the more nonhomologous subunits that assemble together within a complex, the more flexible those subunits tend to be. Importantly, these findings suggest that subunit flexibility should be closely related to the evolutionary history of a complex. We confirm this by showing that evolutionarily more recent subunits are generally more flexible than evolutionarily older subunits. Finally, we investigate the very different explorations of quaternary structure space that have occurred in different evolutionary lineages. In particular, the increased flexibility of eukaryotic proteins appears to enable the assembly of heteromeric complexes with more unique components.

## Abstract 2

The flexibility of individual proteins aids their evolutionary recruitment into complexes with increasing numbers of distinct subunits.

## Author Summary

Proteins often interact with other proteins and assemble into complexes. Here we show that the flexibility of individual proteins is important for their recruitment to complexes, as it facilitates the formation of asymmetric interfaces between different subunits. The role of flexibility becomes increasingly important as a greater number of distinct proteins are packed together within a single complex: the more distinct subunits, the more flexible those subunits need to be. A consequence of this is that, when a protein complex gains a new subunit during evolution, the newer subunit will tend to be more flexible than the older subunits. This suggests that we may be able to partially reconstruct the evolutionary history of a protein complex by considering the flexibility of its subunits. We also find that the types of protein complexes an organism forms are closely related to the flexibility of its proteins, with eukaryotic species, and particularly animals, using their increased flexibility to assemble complexes involving more distinct components.

## Introduction

The assembly of proteins into protein complexes is ubiquitous within the cell [1]–[3]. This provides many potential benefits, such as allosteric regulation, co-localization of distinct biological functions, and protection from aggregation or degradation [4]–[6]. Alternatively, protein oligomerization may in some cases result from random mutations combined with neutral drift [7]. The individual polypeptide constituents of a protein complex—that is, the subunits—can be assembled into a wide variety of symmetric and asymmetric quaternary structure topologies [8]–[11]. Recent work has demonstrated the biological importance of the assembly process by showing that many protein complexes assemble via ordered pathways that have a strong tendency to be evolutionarily conserved [12],[13].

The intrinsic flexibility of proteins is intimately related to their assembly into complexes. For example, flexibility is often crucial for binding—either for facilitating the structural changes that are induced upon binding or for allowing the intrinsic fluctuations within the unbound state that enable a conformational selection binding mechanism [14]. The flexibility of the unbound state also generally correlates with the magnitude of binding-induced conformational changes [15],[16]. However, although the role of flexibility in simple binary interactions is becoming quite well understood, there has been little investigation into how subunit flexibility relates to the diversity of observed quaternary structure topologies. How does flexibility facilitate the assembly of multiple proteins into a protein complex? And given that quaternary structures can evolve in a process analogous to assembly [12],[13],[17], has flexibility been important for this evolution?

The structures of a huge number of protein complexes are now available. Although many structure-based methods are available for characterizing protein flexibility and dynamics, we are primarily interested in the intrinsic flexibility of monomers before they assemble into a complex. Because there are no unbound-state structures available for most individual proteins observed as subunits of protein complexes, it has previously been difficult to characterize their flexibility. Algorithms for predicting intrinsic disorder from protein sequences can provide some useful information, and have revealed a significant tendency for the subunits of large multiprotein complexes to be disordered in isolation [18]–[20].

We recently introduced a simple method for predicting the intrinsic flexibility of proteins from their structures. This method relies on the fact that the folding of a protein from its unfolded state is driven primarily by the intramolecular burial of surface area [21]. Proteins that bury less surface area within their folds will tend to retain more conformational entropy and be more flexible [22]. Therefore, a simple proxy for surface-area burial, the relative solvent-accessible surface area (*A*_rel_), is highly predictive of various flexibility measures, including those calculated from protein structures and those derived directly from experimental measurements [22]. In fact, the correlation between *A*_rel_ and independent measures of flexibility is as strong or stronger than the correlation of those different flexibility measures with each other. *A*_rel_ also shows a strong correspondence with the extent of conformational changes that occur upon complex assembly [16] or disassembly [23].

*A*_rel_ is a simple ratio describing how much solvent-accessible surface area a protein is exposing compared to what we expect for a typical folded, monomeric, crystallizable protein of the same molecular weight. Roughly speaking, *A*_rel_ values of 0.8–0.9 are observed for the most compact, rigid proteins, whereas *A*_rel_ values greater than 1.2 are seen for highly flexible proteins that undergo large conformational changes upon binding [16].

Although *A*_rel_ involves major simplifications, it is important to emphasize that its use as a measure of flexibility arises from fundamental energetic principles—it is not merely a probe of globularity. In fact, some proteins are highly efficient at burying enough intramolecular surface area to become quite rigid, while retaining fairly extended overall conformations. As discussed previously, by assuming constant energy per unit of surface area buried, *A*_rel_ can be directly related to the difference in conformational entropy with respect to an idealized folded state [22]. Furthermore, its remarkable agreement with various computational and experimental flexibility measures strongly supports its utility for large-scale analyses.

There is another major benefit for our purposes here: when *A*_rel_ is calculated for the bound subunits of protein complexes (i.e., by considering the subunits in isolation, ignoring any interfacial contacts), there is a very strong correlation between the *A*_rel_ values of bound subunits and those same proteins in their unbound states [16]. This is illustrated here in Figure S1A. Crucially, this means that the conformation of a protein subunit in its bound state can be used to predict its flexibility in its unbound, monomeric state.

The highly flexible proteins identified with this method also show some correspondence with intrinsic disorder: protein subunits predicted to be disordered in isolation tend to have substantially higher *A*_rel_ values [16],[24]. Furthermore, although the overall sequence determinants of intrinsic disorder are quite different from *A*_rel_ [22], there is still a significant correspondence between the *A*_rel_ values of bound subunits and the fraction of residues predicted to be disordered (Figure S1B). In essence, it appears that *A*_rel_ is able to capture the entire spectrum of protein flexibility associated with binding, of which intrinsic disorder represents one extreme end [25].

It should be noted that, with an approach like this, it can be difficult to distinguish between scenarios where flexibility itself is required for assembly, as opposed to flexibility being a consequence of the structural requirements of a protein complex. For example, proteins that form larger intersubunit interfaces have less surface area available to bury intramolecularly, and are therefore likely to be more flexible in isolation. Similarly, proteins with more elongated shapes will generally be more flexible, and so it may not be possible to differentiate a conformational necessity for elongated shapes within the complex from a requirement for intrinsic subunit flexibility.

In this study, we have used *A*_rel_ to quantitatively investigate the relationships between intrinsic subunit flexibility and the structure, assembly, and evolution of protein complexes. We find that subunit flexibility is strongly associated with the formation of heterologous interfaces required for the assembly of asymmetric, cyclic, and heteromeric complexes. This has major implications for understanding the evolution of protein complexes, as it means that subunit flexibility is often reflective of their evolutionary histories. Moreover, this relationship between flexibility and assembly is also manifested in the very different evolutionary explorations of quaternary structure space observed for prokaryotes and eukaryotes.

## Results and Discussion

### Cyclic and Asymmetric Homomers Are Associated with Increased Subunit Flexibility

**Figure 1:**
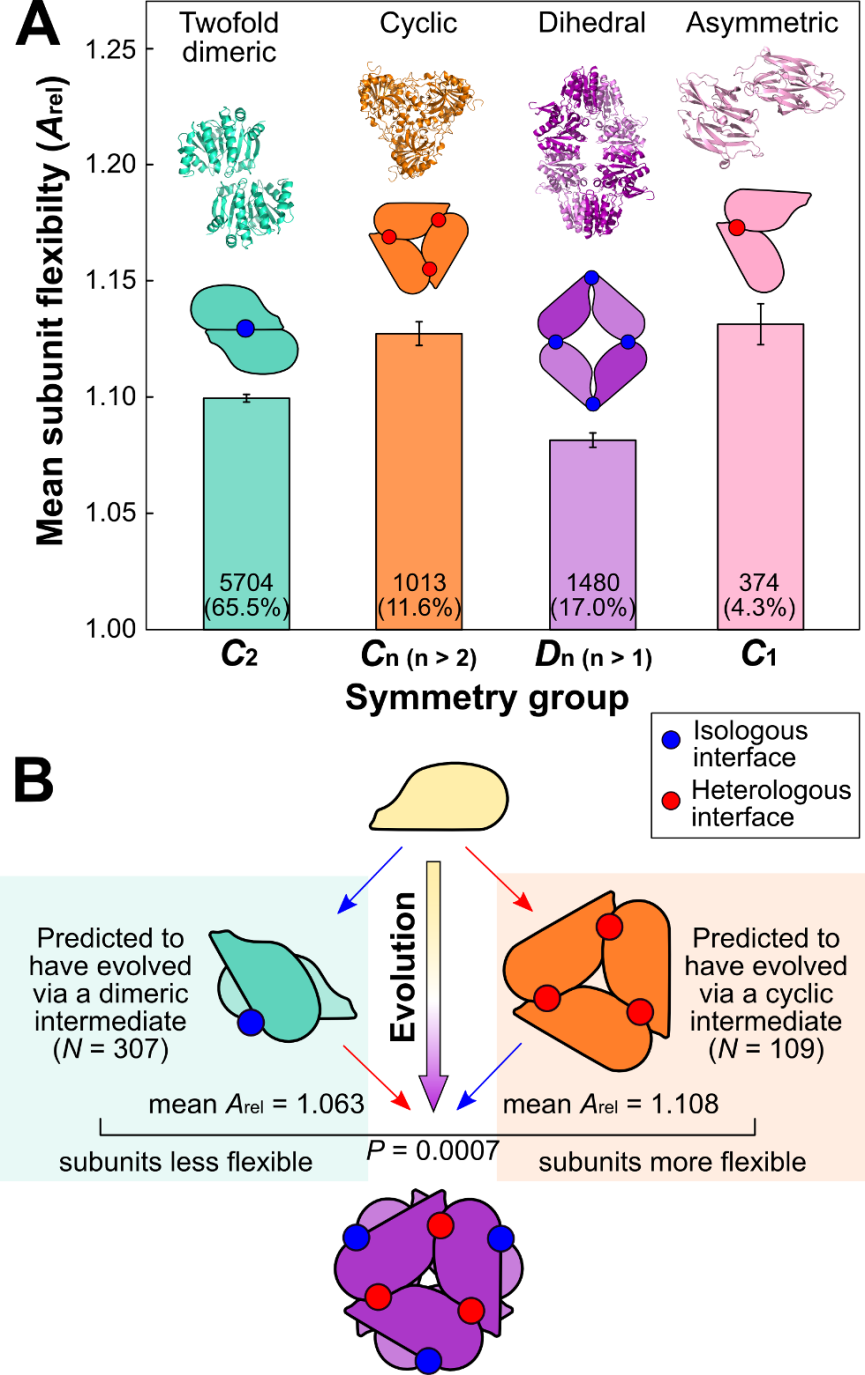
Relating the flexibility of homomeric subunits to quaternary structure topology and evolution. (A) Comparison of subunit flexibility, as measured by *A*_rel_, for homomers from different symmetry groups. An example from each symmetry group is shown above. The numbers and percentages of each group within the total set of homomeric complexes are shown on the bars. These groups comprise all complexes in the PDB except the rare cubic (0.9%) and helical (0.6%) symmetry groups. Error bars represent SEM. Boxplots for each group along with the *p* values between groups are provided in Figure S2A. (B) There are two possible evolutionary pathways for a dihedral hexamer (*D*_3_): via a twofold dimer (*C*_2_) intermediate (left) or via a cyclic (*C*_3_) intermediate (right). When considering all such complexes where two different evolutionary pathways are possible, we observe a strong tendency for those that evolved via a cyclic intermediate to have more flexible subunits. Interestingly, the subunits of complexes with predicted dimeric intermediates are less flexible than those from twofold dimeric complexes (*A*_rel_ = 1.063 versus 1.099, *p* = 5×10^−7^, Wilcoxon rank-sum test) and those from complexes with predicted cyclic intermediates are less flexible (but not significantly so) than those from cyclic complexes (*A*_rel_ = 1.108 versus 1.127, *p* = 0.7). One potential explanation for this is that lower subunit flexibility might be associated with a greater propensity for evolving higher order quaternary structures via dimeric or cyclic intermediates.

We first consider simple homomeric complexes, which are comprised of just a single type of self-interacting subunit. To investigate the relationship between flexibility and symmetry, we group the homomers into the following major classes:

(1) Twofold dimeric complexes, represented by the *C*_2_ symmetry group, are characterized by a single twofold axis of rotational symmetry, which results in an isologous (i.e., symmetric or head-to-head) interface between the two subunits. Such isologous interfaces are extremely common, which has been suggested to be due to their inherent energetic favourability [26],[27].

(2) Cyclic complexes, represented by the *C*_n (n>2)_ symmetry groups, possesses higher order rotational symmetry, with the subunits forming closed rings via heterologous (i.e., asymmetric or head-to-tail) interfaces. Note that although the *C*_2_ complexes do have twofold rotational symmetry, here we will only refer to complexes with at least threefold symmetry as cyclic, due to their distinct interface properties.

(3) Dihedral complexes, represented by the *D*_n (n>1)_ symmetry groups, can be thought of as a doubling of the other topologies through the addition of a new twofold rotational axis (e.g., dimerization of *C*_3_ gives *D*_3_). All dihedral complexes therefore have isologous interfaces corresponding to this twofold axis. Dihedral complexes with at least six subunits usually (but not always) have a mixture of both isologous and heterologous interfaces. Dihedral complexes appear to be particularly conducive to facilitating allosteric regulation, as the isologous interfaces associated with the twofold axis provide a simple way to transmit conformational changes between subunits [9].

(4) Asymmetric complexes, represented by the trivial symmetry group *C*_1_, can be formed in various ways but are characterized by the existence of different subunits in nonequivalent positions (e.g., the asymmetric dimer shown in Figure 1A in which a heterologous interface involving two distinct surfaces is formed).

In Figure 1A, we compare the mean flexibilities, as measured by *A*_rel_, of homomeric subunits from these different groups. Most strikingly, we observe a highly significant tendency for the subunits of cyclic and asymmetric complexes to be more flexible than those forming twofold dimeric or dihedral topologies. Much weaker trends are observed if sequence-based intrinsic disorder predictions are used instead of *A*_rel_ (Figure S3A). Furthermore, when we group the homomers from different symmetry classes by total number of subunits, we observe very little correspondence with subunit flexibility (Figure S4).

What is the origin of this relationship between flexibility and symmetry? A possible explanation is that both cyclic and asymmetric complexes are associated with heterologous intersubunit interfaces involving two distinct surfaces. When forming an asymmetric, heterologous interface, it is easy to imagine how flexibility could be highly beneficial, as it allows for conformational changes of one surface with respect to the other, thus enabling tight intersubunit packing.

In contrast, twofold dimeric and dihedral homomers form isologous interfaces involving self-complementary surfaces. A basic property of an isologous interface is that any conformational change that occurs on one side of the interface must also occur on the other, in order to preserve interface symmetry. This general requirement for equivalent conformational changes on both halves of an isologous interface is likely to make intrinsic flexibility much less advantageous. Therefore, we hypothesize that a major role of subunit flexibility is to facilitate the conformational changes required for heterologous interfaces.

Increased flexibility and conformational changes upon binding are also known to be associated with larger interfaces [16],[28],[29]. This concept is especially intuitive when using *A*_rel_ as a measure of flexibility, as flexible proteins that bury less intramolecular surface area will have more surface available to participate in intermolecular interactions. Thus, one might hypothesize that the increased flexibility associated with asymmetric and cyclic quaternary structures could arise from a requirement for larger interfaces. However, we show in Figure S5 that the symmetry groups associated with increased subunit flexibility do not show a similar association with larger interfaces.

Previously we noted that flexibility shows a significant correspondence with secondary structure: α proteins tend to be more flexible than β proteins [22]. Therefore, in Table S1 we demonstrate that the trends observed here are consistent across different secondary structure classes.

### Subunit Flexibility Reflects the Evolutionary Histories of Homomeric Complexes

The diverse quaternary structures observed in nature are not independent of each other: homomers can evolve from one topology to another [7],[12],[30]. Previously it has been shown that the relative sizes of a homomer's interfaces can be used to predict its evolutionary history, as the largest interface will nearly always have formed first [12],[31]. This means there are multiple possible evolutionary pathways when considering certain quaternary structure topologies. For instance, although all cyclic complexes have exclusively heterologous interfaces and all dihedral complexes have some isologous interfaces, dihedral complexes with at least six subunits can simultaneously have both isologous and heterologous interfaces. In cases where the isologous interfaces are the largest in the complex, the complex will be predicted to have evolved via a dimeric intermediate (Figure 1B, left pathway). On the other hand, if a heterologous interface is the largest, the complex will almost certainly have evolved via a cyclic intermediate (Figure 1B, right pathway).

We considered those homomers with both isologous and heterologous interfaces that therefore have at least two possible evolutionary pathways. These were split into those predicted to have evolved via either twofold dimeric (*C*_2_) or cyclic (*C*_n (n>2)_) intermediates. Interestingly, complexes with dimeric intermediates are nearly three times as abundant as those with cyclic intermediates, consistent with the finding that isologous interfaces are generally more ancient [31],[32], and therefore would be expected to be larger.

We also observe a significant tendency for subunits that assemble via cyclic intermediates to be more flexible than those that assemble via dimeric intermediates (mean *A*_rel_ = 1.108 versus 1.063, *p* = 0.0007, Wilcoxon rank-sum test). In other words, those complexes in which a heterologous interface is the largest will tend to have more flexible subunits, further demonstrating the relationship between subunit flexibility and heterologous interface formation. This also reveals a fascinating connection between subunit flexibility and evolutionary history: just as the evolution of a complex is related to the sizes of its interfaces, it is also reflected in the flexibility of its subunits.

Finally, it is interesting to specifically consider those dihedral complexes predicted to have evolved via dimeric intermediates. If we consider each dimeric precursor together as an individual “subunit,” we can calculate an *A*_rel_ value for the dimer, just as we would for an individual subunit. Given that increased flexibility of individual subunits is associated with assembly into cyclic complexes, we might expect the dimeric precursors of *D*_n (n>2)_ complexes (e.g., trimers or tetramers of dimers) to have higher *A*_rel_ values than those from *D*_2_ (i.e., dimer of dimers) complexes. However, the *A*_rel_ values from the two groups of dimeric precursors are nearly identical (1.086 for *D*_n (n>2)_, 1.088 for *D*_2_, *p* = 0.5, Wilcoxon rank-sum test), suggesting that flexibility at the level of dimeric subcomplexes is not as closely related to quaternary structure as is monomer flexibility.

### Flexibility Enables Packing of Distinct Heteromeric Subunits

Although homomeric interfaces between identical chains can either be isologous or heterologous, heteromeric interactions between dissimilar subunits are inherently heterologous. Therefore, just as flexibility appears to facilitate the packing of heterologous homomeric interfaces, flexibility might also promote the formation of heterologous interfaces in heteromers.

**Figure 2:**
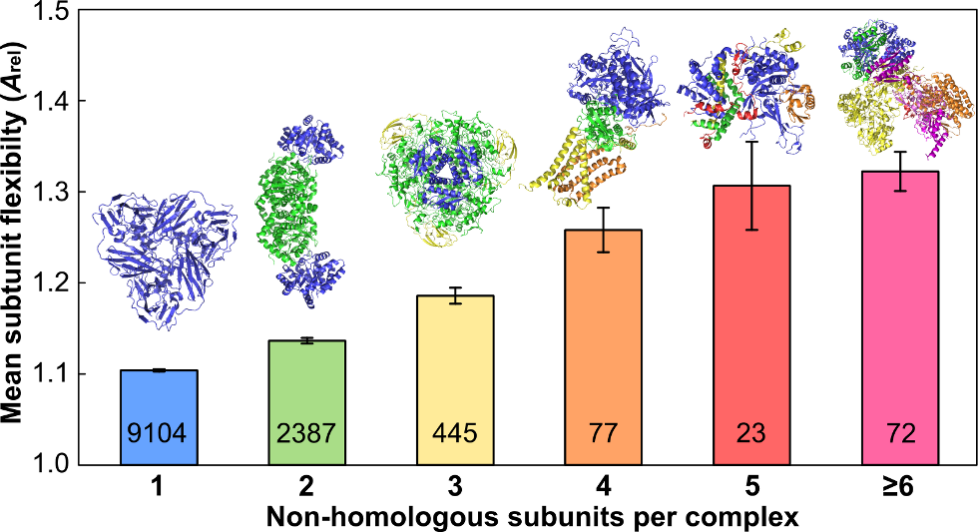
Comparison of subunit flexibility from protein complexes with varying numbers of nonhomologous subunits. Examples of complexes with varying numbers of nonhomologous subunits are shown above. The numbers of unique chains in each group are shown on the bars. Error bars represent SEM. Boxplots for each group are provided in Figure S2B.

To address this, we group protein complexes by their total number of nonhomologous subunits and plot the mean subunit flexibility as measured by *A*_rel_ (Figure 2). In this figure, homomers and homologous heteromers (i.e., heteromers where all the distinct chains are homologous) are represented by a single column (blue), whereas other heteromers can have varying numbers of nonhomologous subunits. There is a very striking association between subunit flexibility and an increasing number of nonhomologous subunits per complex, thus confirming the importance of flexibility in heteromer assembly.

Despite this strong trend, it should be noted that not all subunits of large multiprotein complexes are highly flexible. Although flexibility appears to be important for assembling multiple subunits of different shapes within a single complex, not all subunits need be flexible to achieve this packing. For instance, of those heteromers with four nonhomologous subunits, 13/19 have at least one subunit with *A*_rel_<1.1.

Previously, it was noted that protein complexes with more distinct components tend to be enriched in intrinsic disorder [19]. Here, although we observe a slight tendency for predicted disorder to increase in heteromeric complexes (Figure S3C), the trend is much stronger with *A*_rel_. This further suggests that a range of protein flexibility, of which intrinsic disorder forms part, is important for assembly.

### Flexibility Facilitates the Evolution of New Heteromeric Subunits

**Figure 3:**
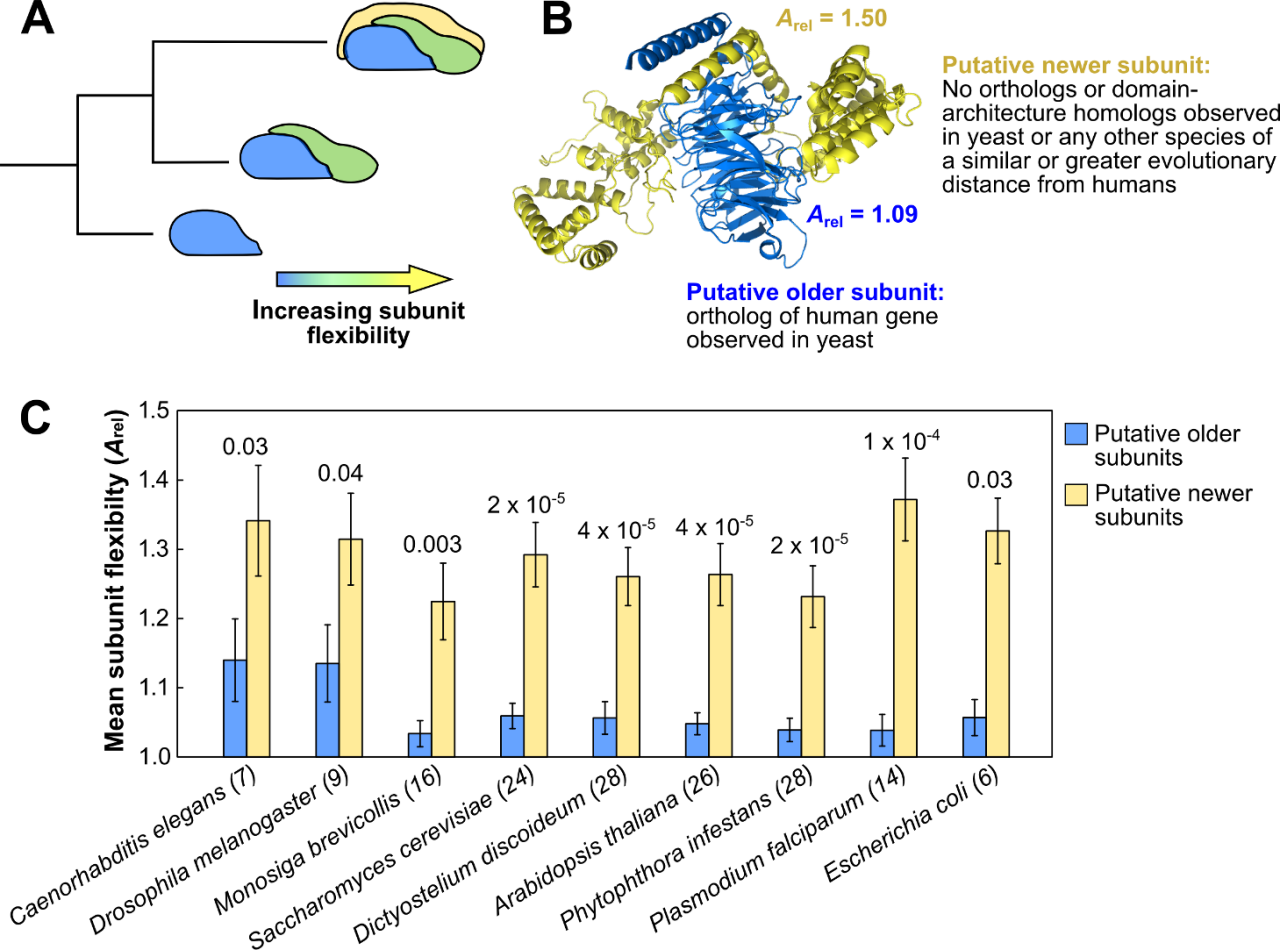
The importance of protein flexibility for the evolution of new heteromeric subunits. (A) Model for the evolution of heteromeric complexes in which new subunits of increasing flexibility are sequentially gained. (B) Example of a protein complex (Gβ5-RGS9, PDB ID: 2PBI) in which different relative ages can be assigned to different subunits. There is an ortholog of Gβ5 (blue) in yeast, whereas no orthologs or domain-architecture homologs of RGS9 (yellow) can be detected in yeast or any other species of a similar or greater evolutionary distance from humans (the most distant ortholog is observed in *Caenorhabditis elegans* and the most distant homolog sharing the same domain architecture is seen in the single-celled eukaryote *Capsaspora owczarzaki*, which is more closely related to humans than yeast). (C) Pairwise comparisons of the flexibility of putative older and putative newer subunits of human (or closely related) protein complexes, with respect to different species. No species more closely related to humans than *C. elegans* and *Drosophila melanogaster* are shown as there are none where >5 complexes with putative older and newer subunits can be identified. The full set of species considered is provided in Table S2. The *p* values calculated with the Wilcoxon signed-rank test are shown for each species, and the numbers of complexes from each species are shown in parentheses. Error bars represent SEM.

The above results have major implications for our understanding of quaternary structure evolution. If we consider a simple scenario in which a heteromer evolves in a sequential manner, gaining a new subunit with each step, then the simplest way to account for this would be if the newly added subunits are more flexible than those from the ancestral complex. This is illustrated in Figure 3A. A similar model was anticipated by Hegyi et al., who suggested that the propensity for intrinsic disorder should be greater in evolutionarily more recent subunits due to the increased disorder propensity in complexes with many subunits [19].

Do the evolutionarily more recent subunits of protein complexes have a significant tendency to be more flexible? To test this, we employed a comparative genomic approach in an attempt to partially reconstruct the evolutionary histories of human heteromers. If an ortholog of a human gene encoding a protein subunit is present in the genome of a given species, then we can assume that that protein was present in the last common ancestor with humans. Of course, the presence of orthologs in an ancestral species does not necessarily mean they interacted [33]–[38]. However, when orthologs of different subunits of the same human complex are present in yeast, the vast majority also form a complex in yeast [39]. Therefore, using the orthologs present in different species taken from the Ensembl Compara [40] and OMA [41] databases, we can say with strong confidence that certain subunits of protein complexes are highly likely to have been present in an ancestral species.

Although we can identify the presence of some subunits in ancestral species with relative simplicity, it is much more difficult to conclusively show that a given subunit was not present, even if no ortholog is detected. For example, the identification of orthologs can be complicated by genome annotation errors or fast evolutionary divergence rates. Moreover, genes can be lost in evolution, so the absence of a gene does not mean that it was not present in an ancestral species. To compensate for these complications, we employed an extremely conservative approach to the identification of subunits that were likely absent in an ancestral species. For each human subunit, we identified the evolutionarily most divergent species in which it might possibly have been present. This was done by considering not just orthologs, but also homologous proteins that share the same domain architectures. These can be of much greater sequence divergence than simple orthologs. Thus, if any ortholog or domain-architecture homolog of a human subunit is present in a given species, we presume that it might possibly (but not necessarily) have formed part of a similar complex in the last common ancestor.

Combining these two approaches, we considered each human (or closely related) protein complex from the perspective of different species of varying evolutionary relatedness to humans. Proteins for which an ortholog could be identified in a given species were considered to be the “putative older subunits.” In contrast, proteins for which no ortholog or homolog could be detected in that species, or any other species of similar or greater evolutionary divergence from humans, were considered to be the “putative newer subunits.” An example of a complex in which two subunits could be confidently assigned as having different evolutionary ages is shown in Figure 3B.

In Figure 3C, we compare the flexibilities of the putative older and newer subunits for several species (all species are provided in Table S2). In this analysis, only those complexes in which both older and newer subunits could be identified were considered. For nearly all species, there is a very strong tendency for the newer subunits to be more flexible than the older subunits, thus supporting our hypothesis that subunit flexibility reflects the relative evolutionary age of subunits.

We can also combine the observations made for different species into a nonredundant set of 61 complexes where both older and newer subunits can be identified. In this case, the newer subunits are also far more flexible than the older subunits (*A*_rel_ = 1.213 versus 1.082, *p* = 6×10^−6^, Wilcoxon signed-rank test). Similarly, in the large majority of complexes (48/61), the newer subunit(s) are more flexible than the older subunit(s) (*p* = 8×10^−6^, binomial test).

Although many subunits from protein complexes of known structure are truncated forms of full proteins (e.g., individual domains), a strong tendency for newer subunits to be more flexible is still observed when only full-length or nearly full-length proteins are considered (*A*_rel_ = 1.245 versus 1.115, *p* = 0.007, *N* = 19). It has also been observed that evolutionarily newer proteins are generally shorter than older proteins [42],[43]. If shorter proteins tended to be more flexible, this could influence our results. However, we find that even when we consider only those cases where the putative newer subunits are longer than the older subunits, the newer subunits are still more flexible (*A*_rel_ = 1.221 versus 1.115, *p* = 0.007, *N* = 24).

An additional concern is that some fast-evolving proteins may have diverged beyond detectable homology, yet still share structural and functional similarity and possibly still interact within the same complex. If there existed a tendency for more flexible proteins to evolve at a faster rate, then more flexible proteins might simply appear to be more recent due to their lower conservation. Generally it is thought that, although the more flexible regions of a given protein tend to evolve more quickly than its more rigid regions, there is little correspondence between flexibility and evolutionary conservation at the global protein level [17]. We address this further in Figure S6, showing that there is no clear propensity for evolutionarily newer proteins to be more flexible overall (i.e., when not considered at the individual complex level), although there is a slight tendency for the most highly flexible proteins to be less conserved.

Finally, there is a completely different way by which we can assess the propensity for evolutionarily more recent subunits to be more flexible. As an alternative to the scheme in Figure 3A, we can hypothesize that existing subunits might have evolved to become more flexible in order to accommodate new, more rigid subunits. To address this, we “normalize *A*_rel_” for the variation that occurs between homologous proteins that form subunits of different complexes, and for the variation that occurs between evolutionarily unrelated protein families (Figure S7). This analysis shows that very little of the trend in Figure 2 can possibly be explained by increasing flexibility of existing subunits, thus strongly supporting the scenario in Figure 3A.

### Evolutionary Exploration of Quaternary Structure Space Is Related to Proteome Flexibility

**Figure 4:**
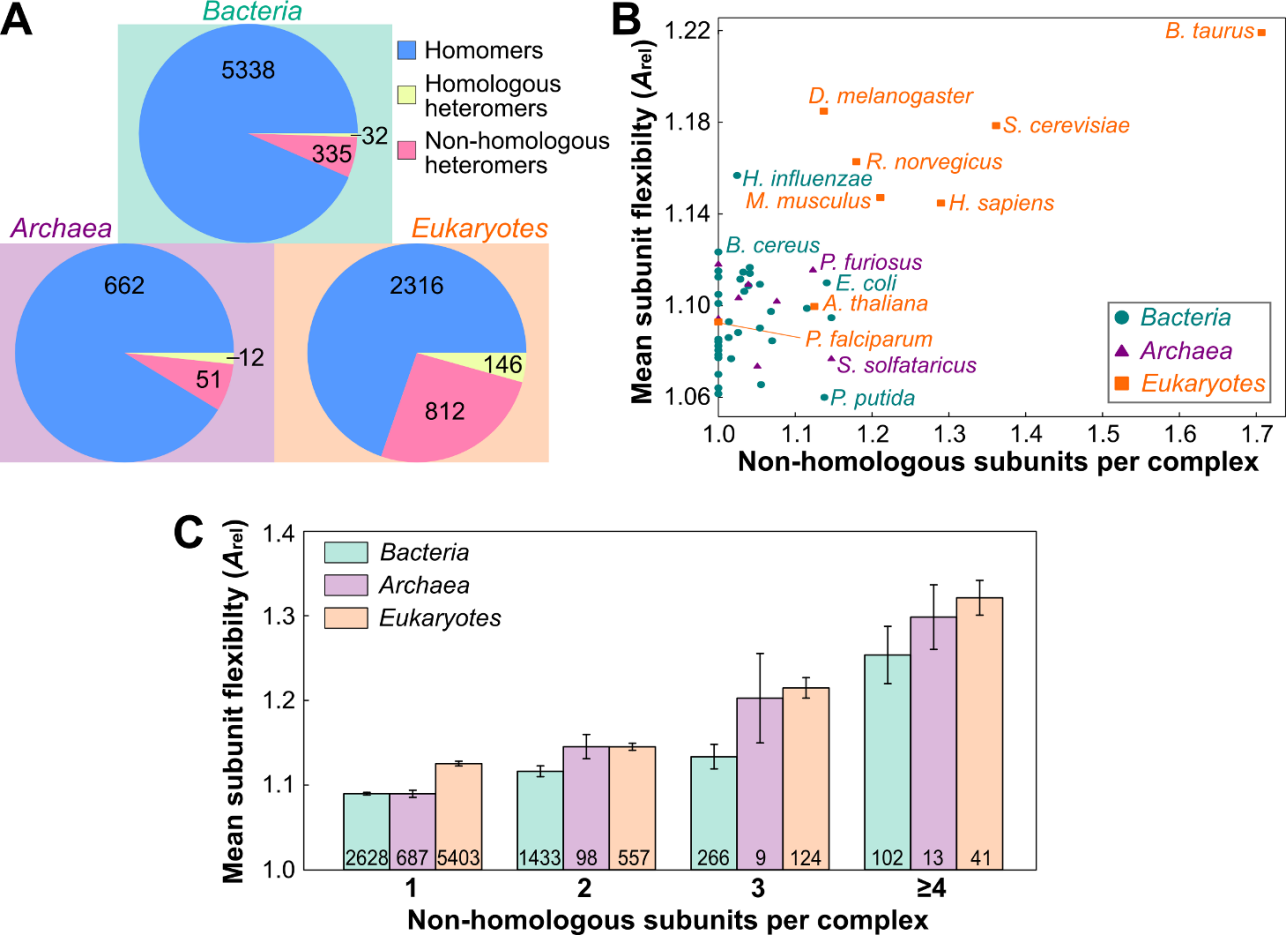
The relationship between evolution, quaternary structure topology, and protein flexibility. (A) Comparison of the numbers of homomers, homologous heteromers (i.e., heteromers where all distinct chains are homologs), and nonhomologous heteromers from bacteria, archaea, and eukaryotes. (B) Comparison of the mean subunit flexibility and number of nonhomologous subunits per complex for the 50 species with the most complexes in our dataset. Values for all species are provided in Table S3. (C) Comparison of subunit flexibility for protein complexes with varying numbers of nonhomologous subunits from bacteria, archaea, and eukaryotes. Error bars represent SEM. A similar species-level analysis is provided in Figure S8.

The observation that subunits gained later in evolution tend to be more flexible raises interesting questions about proteome and interactome evolution. Specifically, it suggests that the average flexibility of proteins in an organism might increase over the course of evolution as new proteins are acquired and the number of protein complex interactions increases. Therefore, it is interesting to first consider how quaternary structure varies in evolution, by comparing the proportion of homomeric and heteromeric complexes in bacteria, archaea, and eukaryotes (Figure 4A). Interestingly, a far greater percentage of eukaryotic complexes in our dataset are heteromeric (29.3%), as compared to bacterial (6.4%) or archaeal (8.7%) complexes (*p*<10^−34^, Fisher's exact test). This is consistent with the previous observation that heteromers are enriched in vertebrates relative to unicellular organisms [44]. Although gene duplications in eukaryotes are known to have resulted in many homologous heteromers [45], these still comprise only a small fraction of the total heteromers (Figure 4A). These huge differences strongly suggest that heteromeric topologies are much more frequently utilized in eukaryotes than prokaryotes. Moreover, this is compatible with the fact that eukaryotes also generally have larger genomes. The larger number of protein-coding genes therefore provides more different proteins with which to form complexes.

Next, to explore the evolutionary relationship between flexibility and quaternary structure, we grouped complexes by their species of origin and plotted the number of nonhomologous subunits per complex against the mean subunit flexibility (Figure 4B; values for all species provided in Table S3). There is a striking distinction between prokaryotes and eukaryotes: the eukaryotes tend to have more flexible subunits that form complexes with more unique components, whereas bacterial and archaeal complexes have fewer, less flexible subunits. Although there are certainly some biases in the complexes crystallized from different species, the consistency of the division between prokaryotes and eukaryotes suggests that it is reflective of real evolutionary differences.

There are two eukaryotes that cluster with the prokaryotes: the plant *Arabidopsis thaliana* and the protozoan *Plasmodium falciparum*. This is quite interesting given that these two species are the most evolutionary divergent eukaryotes, relative to the more closely related yeast and metazoans [46]. When all 174 other plant complexes (excluding *A. thaliana*) are considered together, they have more nonhomologous subunits per complex (1.172) than observed in any of the prokayotes, but very low subunit flexibility (mean *A*_rel_ of 1.067). From this limited evidence, it is difficult to tell whether these results reflect genuine evolutionary differences. However, this does hint that some of this divergence may have occurred in the fungi/metazoa lineage.

The eukaryotic species have a much greater spread in nonhomologous subunits per complex. *Bos taurus*, in particular, has more than any other species. A possible explanation for this is that many of these large multiprotein complexes are likely to have been natively purified from bovine tissues. Thus, the complexes tend to contain more of the biologically relevant subunits present *in vivo*, whereas complexes from other organisms are more likely to have been recombinantly produced. Interestingly, we note that *Saccharomyces cerevesiae* also has a relatively large number of nonhomologous subunits per complex, as does *Escherichia coli* when compared to other prokaryotes. These organisms are often used for protein production and so their complexes may also be more likely to have been natively purified. These results highlight the interesting (albeit probably unsurprising) point that protein complexes *in vivo* are likely to have a much greater tendency to contain more distinct subunits than has generally been observed crystallographically.

Figure 4B suggests that the increase in protein flexibility observed in eukaryotes could possibly be explained by the fact that their protein complexes have more distinct components. Therefore, we next compared the flexibility of subunits from bacteria, archaea, and eukaryotes, while controlling for the number of nonhomologous subunits (Figure 4C). Interestingly, the subunits of eukaryotic complexes still tend to be more flexible than those from bacteria. The archaeal subunits are generally intermediate in flexibility to bacteria and eukaryotes, although there are far fewer archaeal complexes in the dataset. Thus, although increased flexibility in eukaryotes is important for facilitating heteromer assembly, much of the increase in eukaryotic proteome flexibility is clearly independent of the physical requirement for packing multiple subunits within individual complexes. Similar relationships between flexibility and nonhomologous subunits are observed for individual species (Figure S8), which suggests that these results are not influenced by any strong species-level bias.

As a complement to this structure-based analysis of flexibility using *A*_rel_, we also looked at the relationship between predicted intrinsic disorder and protein–protein interactions. Previous observations have shown a strong tendency for proteins with more interaction partners to possess a greater fraction of intrinsically disordered residues [47]–[49]. This could be considered somewhat analogous to our observation of increased flexibility in complexes with multiple distinct subunits. In Figure S9, we show that this trend is observed for the bacterial, archaeal, and eukaryotic species with the most experimentally identified protein–protein interactions. These nonstructural results are consistent with our structural analysis, emphasizing the importance of flexibility and disorder for facilitating protein interactions across evolution. They also highlight an increased level of intrinsic disorder in eukaryotes that appears to be independent of the number of interactions made.

## Conclusions

In this study, we have demonstrated a close association between intrinsic subunit flexibility and the assembly of protein complexes. The origin of this is simple: because flexibility is largely controlled by how little surface area a protein buries intramolecularly [22], then the more flexible the protein, the more surface area that will be available to participate in intermolecular interactions. This is why increased flexibility, disorder, and conformational changes upon binding are associated with larger interfaces [16],[28],[29],[50]. The evidence presented here suggests that flexibility is particularly conducive to the formation of heterologous interfaces, in which two distinct surfaces interact with each other. Therefore, flexibility appears to facilitate the assembly of asymmetric, cyclic, and heteromeric complexes.

This work also extends our understanding of protein evolution, as it shows how the evolutionary history of a protein complex can be directly related to the flexibility of its subunits. This suggests that flexibility could potentially be quite useful in the reconstruction of protein complex evolutionary histories. To some extent, our results suggest that the eukaryotic increase in flexibility may have been driven by the evolution of protein complexes with more components. In addition, it is possible that some of the increased flexibility in eukaryotic subunits may be reflective of a greater propensity to form multiple nonconcurrent interactions, as has been seen for intrinsic disorder [49],[51],[52]. However, the increase in flexibility might also be related to selection for function other than protein complex assembly, increased tolerance due to compartmentalization and chaperones, or simply genetic drift [53].

This new knowledge of the relationship between quaternary structure topology and flexibility could aid the prediction of protein complex topologies from limited information. For example, if some knowledge of intrinsic flexibility is available (based upon sequence, structure, or experiments), this could be used to help assess the relative likelihoods of different quaternary structure arrangements. Similarly, just as flexibility appears to facilitate quaternary structure evolution, it might also prove important for engineering multiprotein assemblies, if the principles of flexibility and interactions can be harnessed to enable the packing of heterologous interfaces.

In the present study, we have interpreted our results as showing that intrinsic flexibility facilitates the assembly and evolution of quaternary structure. However, it is possible that, rather than flexibility being required for assembly, it can to an extent be thought of as arising from the physical requirements of the bound state. That is, the packing of multiple, different-shaped subunits within a single complex may necessitate flexibility. Any protein that could form sufficient intersubunit interactions might be inherently flexible in its unbound state due to a lack of intramolecular contacts. A related issue has recently been discussed by Janin and Sternberg, who suggested that many intrinsically disordered proteins are simply “proteins waiting for a partner” [54]. They propose that actual disorder should be rare *in vivo*, as these proteins will usually be protected by chaperones prior to assembly. Ultimately, more studies will be required to quantify the extent of *in vivo* flexibility and disorder, and to further disentangle the functional importance of unbound-state properties from the conformational requirements of bound subunits.

## Methods

### Protein Structure Dataset

Biological units of protein crystal structures (<5 Å resolution) were taken from the Protein Data Bank on 2012-08-08, considering chains ≥40 residues. We filtered out backbone-only models and structures containing nucleic acids or >10% nonwater heteroatoms. Heteromers formed by subunit cleavage were also removed by identifying nonidentical chains from the same complex having the same *db_id* assignment. Additionally, protein complexes annotated as having quaternary structure assignment errors [55] were excluded. Symmetry groups were taken directly from the PDB. The number of nonhomologous subunits in a complex was defined on the basis of chains with distinct SUPERFAMILY “family” domain assignments [56]. Complexes in which no subunits had domain assignments were not considered in the “number of nonhomologous subunits” analyses.

Solvent-accessible surface areas and interface sizes were calculated with AREAIMOL. *A*_rel_ values were calculated according to *A*_rel_ = *A*_s_/4.44*M* ^0.77^, where *A*_s_ is the solvent-accessible surface area and *M* is the molecular mass, as in [22]. The *A*_rel_ values of the dimeric precursors of dihedral complexes were calculated in the same way, except the total solvent-accessible surface area of each dimer was calculated, and the masses of the two subunits were summed. Complexes with two possible assembly pathways were identified as those symmetric homomers with at least six subunits having both heterologous and isologous interfaces >800 Å^2^. Homomeric interfaces were identified as being isologous if the correlation between the residue-specific buried surface area for each subunit in an interacting pair was >0.7.

Secondary structure was calculated for each protein chain with STRIDE [57], and the following secondary structure groups were used in Table S1: α proteins (>20% α-helical residues), β proteins (>20% β-strand residues), and αβ proteins (>20% α-helical residues and >20% β-strand residues). Intrinsic disorder was predicted from protein sequences with IUPRED [58], using the “long” setting and threshold of 0.5 for identifying disordered residues.

Protein complexes in which all unique chains share >50% sequence identity were clustered. In addition, to avoid highly similar complexes that vary only slightly in their subunit composition, heteromeric complexes sharing at least four unique chains were clustered. From each cluster, only the complex with the most amino acid residues (ignoring subunit repeats) was selected for the nonredundant set used in this study (8,700 homomers and 1,552 heteromers). However, we note that this sequence-redundancy filtering is not perfect, as proteins can share sequence identity significantly lower than 50%, yet still be quite similar structurally. Therefore, we also created a stricter nonredundant set of protein complexes that are nonhomologous at the structural level by only considering only complexes with unique SUPERFAMILY domain assignments (2,208 homomers and 1,046 heteromers). The main structural analyses from Figures 1A and 2 were repeated with this strict dataset, and the results are essentially the same (Table S1). All complexes used in this study and relevant subunit properties are included in Tables S4 and S5.

### Evolutionary Analysis

To map human genes against protein structures, a *blastp* search against all human proteins in Ensembl was performed for each protein chain. All chains with >70% sequence identity to a human protein were considered. Orthologs of these proteins were then identified in a variety of different species with Ensembl Compara [40] and OMA [41] (all species are listed in Table S6). For some species, both databases were used, whereas some species were only available in one or the other. If an ortholog of a human gene that maps to a protein complex subunit was present in a given species, we presumed that that subunit was present in the last common ancestor with humans, and is therefore a “putative older subunit” with respect to that species. The analysis considering full-length and nearly full-length proteins only included chains where at least 75% of the residues from the full-length protein were observed in the crystal structure.

To identify the “putative newer subunits” that were likely not present in an ancestral species, we also considered homologs at the level of domain architecture. This allows us to identify more divergent proteins that might have possibly been playing a similar subunit role in an ancestral complex. Importantly, we do not use this information to say that an ancestral subunit was present, but instead to say that an ancestral subunit might possibly have been present. Using SUPERFAMILY genome-scale domain assignments [59], we asked for each human subunit whether any protein in a given organism has the same set of domains (ignoring N- to C-terminal order) as the full-length human protein. If so, this subunit was excluded as a “putative newer subunit” with respect to that species. Human proteins with no SUPERFAMILY domain assignments were not considered as either newer or older subunits. Finally, in addition to checking that any ortholog or homologs are not present in a given species, we also checked that they were not present in any species of a similar or greater evolutionary distance from humans. This helps to avoid bias from gene loss and genome annotation errors. The ranked evolutionary distance from humans for each species used for this analysis is provided in Table S6.

To generate nonredundant sets of protein complexes having both putative older subunits and putative newer subunits, we only considered a single complex mapping to a given pair of old and new human genes. Similar filtering was performed when the sets of different species were combined. All the sets of putative older and newer subunits are provided in Table S6. Overall, although they include different species, the Ensembl Compara and OMA databases gave very similar results. Table S2 also includes the results for different species calculated with either one or the other databases.

## Supporting Information

Figure S1 *A*_rel_ values of bound subunits from protein complexes are predictive of intrinsic flexibility in the unbound state. (A) Comparison between *A*_rel_ values of monomeric proteins, *A*_rel_(free), and those same proteins (>98% sequence identity, <2% length difference) bound as subunits within homomeric or heteromeric complexes, *A*_rel_(bound). In total, 288 homomer and 387 heteromer pairs were identified from the nonredundant dataset used in this study (provided in Table S5). The very strong correlations demonstrate that the *A*_rel_ of the bound state is highly predictive of the *A*_rel_, and thus the intrinsic flexibility, of the free state. The mean difference between *A*_rel_(bound) and *A*_rel_(free) is 0.9% (mean absolute difference of 2.6%) for homomers and 0.7% (mean absolute difference of 3.0%) for heteromers, suggesting that there is a very slight tendency for *A*_rel_(bound) to overestimate *A*_rel_(free). These values are consistent with a recent study showing that the accessible surface area of interface residues in the bound state are on average 3.3% higher than in the unbound state [60]. The outliers here are mostly from domain-swapped homomers, where the swapped bound state will have a substantially higher *A*_rel_ value, but the free state is stabilized by the same intermolecular interactions being formed intramolecularly. Given the overall high correlations and the rarity of outliers observed here, and the fact that domain swapping is only observed in ∼5% of protein families [61], the effect of domain swapping on our analyses should be minimal. (B) Fraction of predicted intrinsically disordered residues for bound subunits for which no corresponding monomer structure exists, grouped by *A*_rel_ value. Error bars represent SEM. The overall correlation (*r*) between *A*_rel_ and intrinsic disorder is 0.313 (*N* = 9,527). For those subunits for which a corresponding monomer structure does exist (sequence identity >50%), the correlation is much lower (*r* = 0.137, *N* = 2,695).
(TIFF)

Figure S2 Boxplot representations of *A*_rel_ distributions for subunits from different groups of protein complexes. Boxplots are generated in R using standard settings. The *y*-axes are plotted logarithmically. Nonoverlapping notches can be used as a rough indicator of statistically significant differences between two groups. (A) Subunits of homomers from different symmetry groups, as in Figure 1A. The *p* values for the differences between groups are shown calculated with the Wilcoxon rank-sum test. (B) Subunits from heteromers with different numbers of nonhomologous subunits, as in Figure 2.
(TIFF)

Figure S3 Intrinsic disorder is also related to quaternary structure topology, but less so than *A*_rel_ as a measure of intrinsic flexibility. Comparison of the percentage of residues predicted to be intrinsically disordered for subunits from (A–B) homomeric complexes from different symmetry groups (compare to Figure 1A) and (C–D) complexes with different numbers of nonhomologous subunits (compare to Figure 2A). (A) and (C) show means with SEM and (B) and (D) show boxplots, as in Figure S2. The trends for homomers in (A) and (C) mirror the results using *A*_rel_, but are not as strong (compare to *p* values in Figure S2A).
(TIFF)

Figure S4 Subunit flexibility is largely independent of the number of subunits in a homomeric complex. Comparison of subunit flexibility, as measured by *A*_rel_, to the number of subunits in homomers from different symmetry groups. The overall correlations (*r*) between *A*_rel_ and number of subunits are 0.115 for cyclics (*p* = 0.0002), 0.056 for dihedrals (*p* = 0.03), and 0.092 for asymmetrics (*p* = 0.07). Thus, there appears to be a very slight but significant tendency for larger homomers to have more flexible subunits. Error bars represent SEM.
(TIFF)

Figure S5 Interface size is related to symmetry but does not explain the observed flexibility trends. Comparison of interface sizes for homomeric subunits in different symmetry groups: (A) mean interface area per subunit; (B) mean relative interface area per subunit (i.e., what fraction of the surface forms interface). Error bars represent SEM. The trends here show essentially no correspondence with the flexibility results in Figure 1A, demonstrating that the association between flexibility and symmetry is not simply due to a requirement to form larger interfaces.
(TIFF)

Figure S6 The observation that evolutionarily more recent subunits are more flexible does not arise from a general tendency for increased flexibility in newer proteins. Although we observed a strong trend for the evolutionarily more recent subunits of protein complexes to be more flexible, it is possible that this could to some extent reflect a general tendency for evolutionarily more recent proteins to be more flexible. This could also arise if more flexible proteins tend to evolve at a faster rate, thus making them less likely to be detected as orthologs. We have addressed this in two ways: (A) comparison of *A*_rel_ values for human (or closely related) subunits whose most ancient orthologs are of varying evolutionary ages. Error bars represent SEM. There is no clear tendency for newer subunits to be more flexible (although subunits conserved in bacteria do appear to be less flexible), suggesting that our results cannot be explained by a general tendency for newer proteins to be more flexible. Full species names and the different evolutionary groups are provided in Table S6. (B) Comparison of sequence identities for subunits of varying flexibility. Here we grouped subunits by *A*_rel_ and plotted the mean sequence identities of Ensembl Compara orthologs from different species. This shows that, for the most part, sequence conservation is fairly constant with respect to *A*_rel_, although there is some tendency for the most flexible human subunits to be less conserved, particularly when compared to yeast.
(TIFF)

Figure S7 The correspondence between subunit flexibility and the number of nonhomologous subunits per complex is not due to existing subunits evolving to become more flexible. The correspondence between subunit flexibility and the number of nonhomologous subunits per complex could possibly be explained if the existing (i.e., older) subunits of a complex can evolve to become more flexible as new, more rigid subunits are added. To test this, we grouped subunits by their SUPERFAMILY domain architecture. We considered only those groups where evolutionarily related proteins participate in different complexes that have different numbers of nonhomologous subunits. We then plot the relationship between *A*_rel_ and the number of nonhomologous subunits in three ways (values provided in Table S7): (A) The blue bars are essentially equivalent to Figure 2, although only those subunits that are also considered in (B) and (C) are included here. (B) The pink bars represent the “interfamily normalized” *A*_rel_ values, in which all variation should be due to evolutionary changes within a domain family. Here, the *A*_rel_ value for each subunit has been divided by the mean *A*_rel_ value for all subunits with the same domain architecture. The values are then all scaled by the mean *A*_rel_ of all subunits in the dataset. If there is a tendency for evolutionarily related proteins to be more flexible when they are part of complexes with more nonhomologous subunits, then we would expect these values to show an increasing trend. However, there is only a very slight trend, which does not explain the variation shown in (A). (C) The yellow bars represent the “intrafamily normalized” *A*_rel_ values, in which all variation should be due to differences between different types of domains. In these, the *A*_rel_ value of each subunit has been replaced with the mean *A*_rel_ value for all subunits with the same domain architecture. Thus we can see that nearly all of the trend in (A) can be explained by differences between evolutionarily unrelated proteins, strongly suggesting that the scheme in Figure 3A is correct and that existing subunits do not generally evolve to become more flexible in order to accommodate new subunits.
(TIFF)

Figure S8 The association between flexibility and the number of nonhomologous subunits per complex is preserved across different species. This plot is essentially the same as Figures 2 and 4C, except it considered separately the nine species with the most heteromers in our nonredundant dataset. A clear trend is observed for nearly all species. Only *M. musculus* and *T. maritima* appear to deviate, although this is likely due to the limited size of the dataset, including the fact that no complexes with >3 nonhomologous subunits are present for these species.
(TIFF)

Figure S9 Increasing intrinsic disorder is associated with a greater number of interaction partners across different species. Comparison of the percentage of residues predicted to be intrinsically disordered for proteins grouped by their number of experimentally identified interaction partners. Experimental protein–protein interactions were taken from STRING v9.0 [62], using only interactions with an experimental evidence confidence score >0.3. Varying the threshold from 0.15 to 0.7 preserved the same general trends. The bacterial, archaeal, and two eukaryotic species with the most interactions are shown here. Error bars represent SEM.
(TIFF)

Table S1 Controlling for structural factors when comparing the flexibilities of subunits from different groups of protein complexes. This table provides the raw values for the main results in Figures 1A and 2. It also provides the values for these analyses broken down by secondary structure group, and using only the strict structurally nonredundant set of protein complexes, filtered at the domain level.
(XLSX)

Table S2 Pairwise flexibility comparison between putative older and putative newer subunits of protein complexes with respect to all species used in this analysis. These values are the same as used in Figure 3C, except that all species are shown here. We also include the results when only Ensembl Compara or only OMA are used as a source of orthologs.
(XLSX)

Table S3 Comparison of the mean subunit flexibility and number of nonhomologous subunits per complex from different species. These are the same values used in Figure 4B, except that all 263 species with at least five nonredundant complexes in our dataset are shown here.
(XLSX)

Table S4 Homomeric and heteromeric protein complexes used in this study.
(XLSX)

Table S5 Properties of protein complex subunits.
(XLSX)

Table S6 Putative older and newer subunits identified from each species, along with the combined set of nonredundant complexes that have both older and newer subunits. Also included here are the results of the analyses including only full-length or nearly full-length PDB chains, and only complexes in which the newer subunits are longer than the older subunits. The highest sequence identity between a human gene and its ortholog in Ensembl Compara is provided for each older subunit.
(XLSX)

Table S7 *A*_rel_, interfamily normalized *A*_rel_, and intrafamily normalized *A*_rel_ values for subunits from different domain families. These are the values used for the analysis in Figure S7.
(XLSX)

## Abbreviations

*A*_rel_: relative solvent accessible surface area
