# Supplementary figures and images for: Protein Flexibility Facilitates Quaternary Structure Assembly and Evolution
Source: PLoS Biol. 2014 May 27;12(5):e1001870. doi: 10.1371/journal.pbio.1001870 (PMC4035275; doi:10.1371/journal.pbio.1001870)

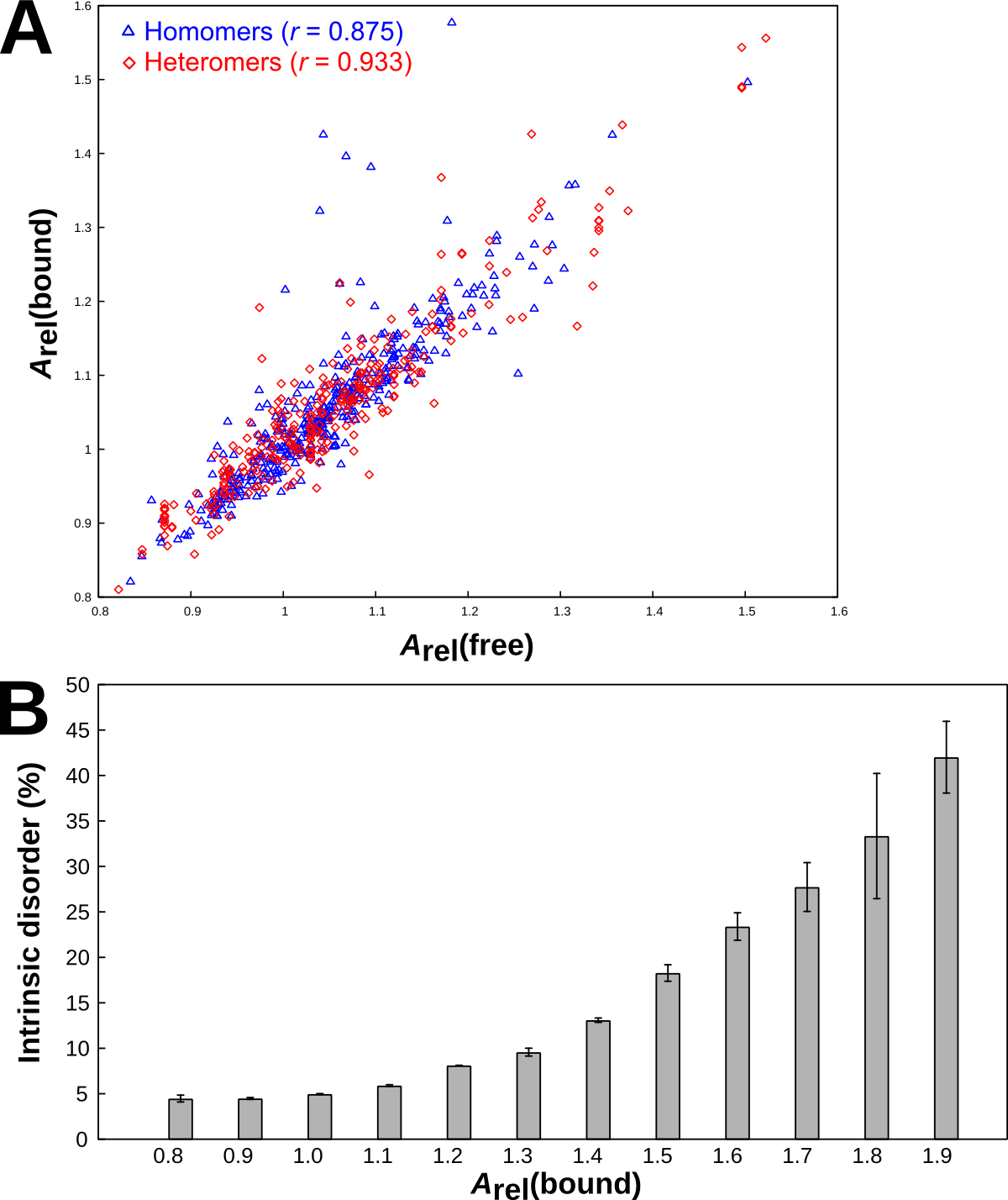

Supplement: Figure S1 — A rel values of bound subunits from protein complexes are predictive of intrinsic flexibility in the unbound state. (A) Comparison between A rel values of monomeric proteins, A rel(free), and those same proteins (>98% sequence identity, <2% length difference) bound as subunits within homomeric or heteromeric complexes, A rel(bound). In total, 288 homomer and 387 heteromer pairs were identified from the nonredundant dataset used in this study (provided in Table S5). The very strong correlations demonstrate that the A rel of the bound state is highly predictive of the A rel, and thus the intrinsic flexibility, of the free state. The mean difference between A rel(bound) and A rel(free) is 0.9% (mean absolute difference of 2.6%) for homomers and 0.7% (mean absolute difference of 3.0%) for heteromers, suggesting that there is a very slight tendency for A rel(bound) to overestimate A rel(free). These values are consistent with a recent study showing that the accessible surface area of interface residues in the bound state are on average 3.3% higher than in the unbound state [60]. The outliers here are mostly from domain-swapped homomers, where the swapped bound state will have a substantially higher A rel value, but the free state is stabilized by the same intermolecular interactions being formed intramolecularly. Given the overall high correlations and the rarity of outliers observed here, and the fact that domain swapping is only observed in ∼5% of protein families [61], the effect of domain swapping on our analyses should be minimal. (B) Fraction of predicted intrinsically disordered residues for bound subunits for which no corresponding monomer structure exists, grouped by A rel value. Error bars represent SEM. The overall correlation (r) between A rel and intrinsic disorder is 0.313 (N = 9,527). For those subunits for which a corresponding monomer structure does exist (sequence identity >50%), the correlation is much lower (r = 0.137, N = 2,695). (TIFF) [file pbio.1001870.s001.tiff]

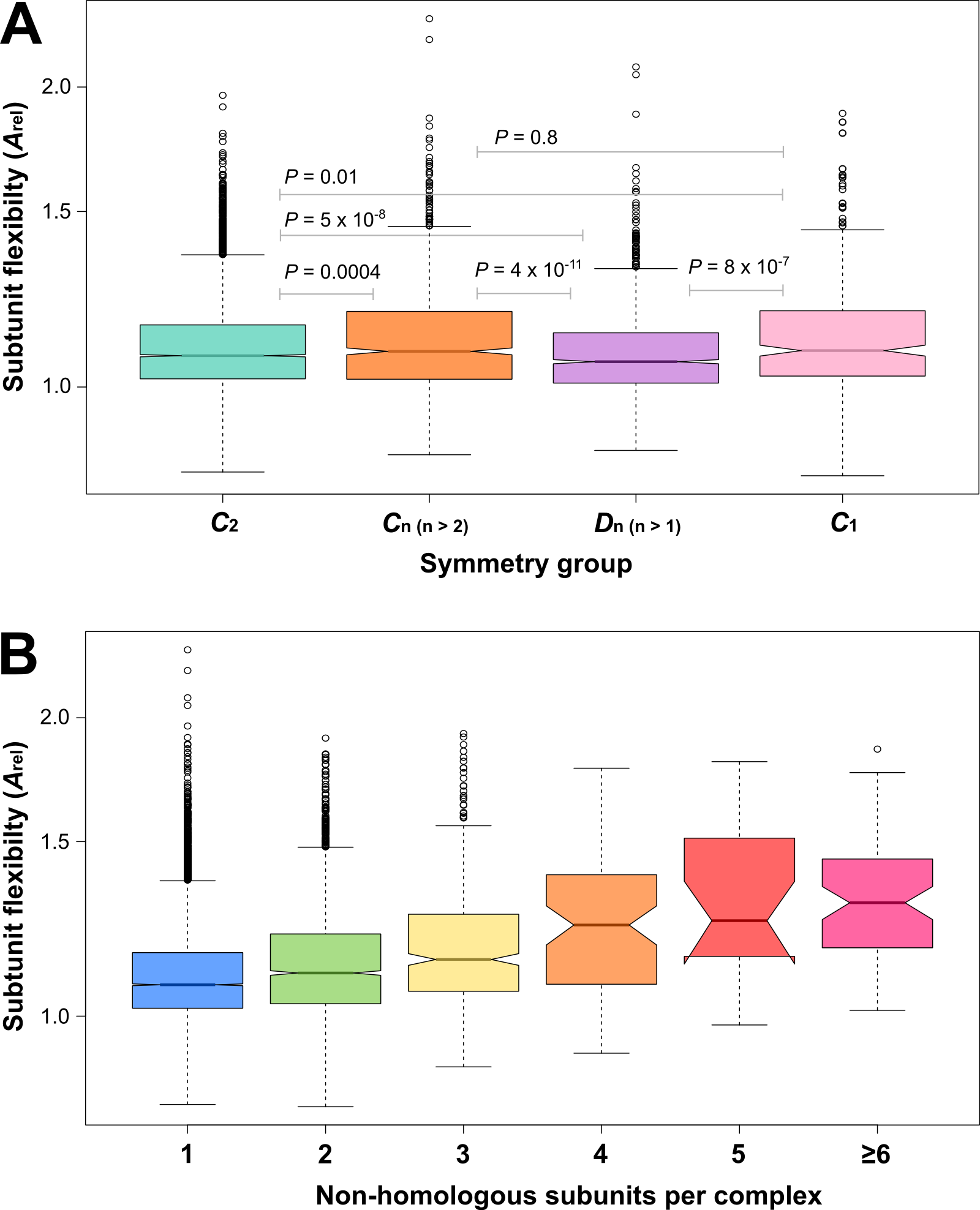

Supplement: Figure S2 — Boxplot representations of A rel distributions for subunits from different groups of protein complexes. Boxplots are generated in R using standard settings. The y-axes are plotted logarithmically. Nonoverlapping notches can be used as a rough indicator of statistically significant differences between two groups. (A) Subunits of homomers from different symmetry groups, as in Figure 1A. The p values for the differences between groups are shown calculated with the Wilcoxon rank-sum test. (B) Subunits from heteromers with different numbers of nonhomologous subunits, as in Figure 2. (TIFF) [file pbio.1001870.s002.tiff]

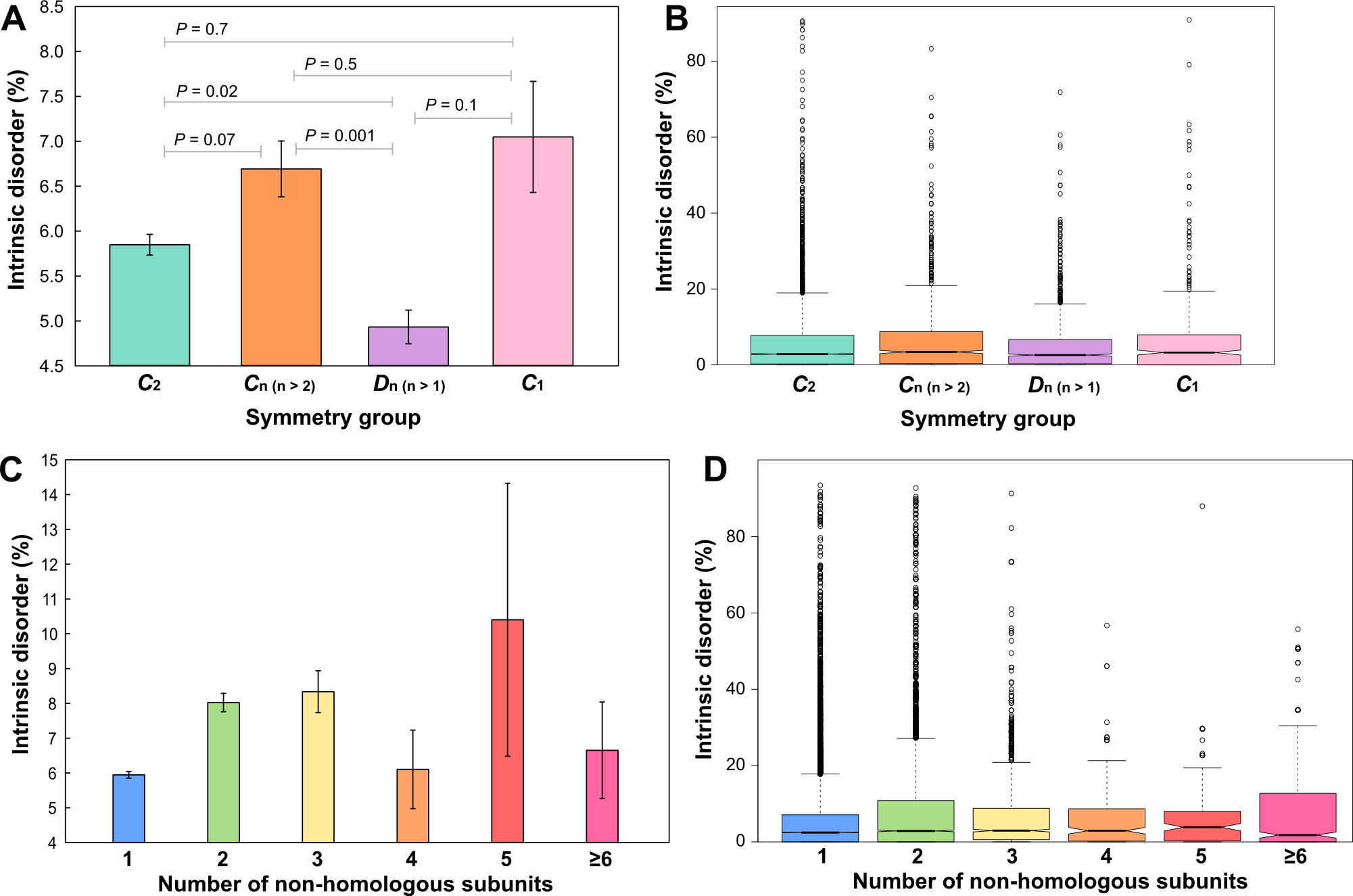

Supplement: Figure S3 — Intrinsic disorder is also related to quaternary structure topology, but less so than A rel as a measure of intrinsic flexibility. Comparison of the percentage of residues predicted to be intrinsically disordered for subunits from (A–B) homomeric complexes from different symmetry groups (compare to Figure 1A) and (C–D) complexes with different numbers of nonhomologous subunits (compare to Figure 2A). (A) and (C) show means with SEM and (B) and (D) show boxplots, as in Figure S2. The trends for homomers in (A) and (C) mirror the results using A rel, but are not as strong (compare to p values in Figure S2A). (TIFF) [file pbio.1001870.s003.tiff]

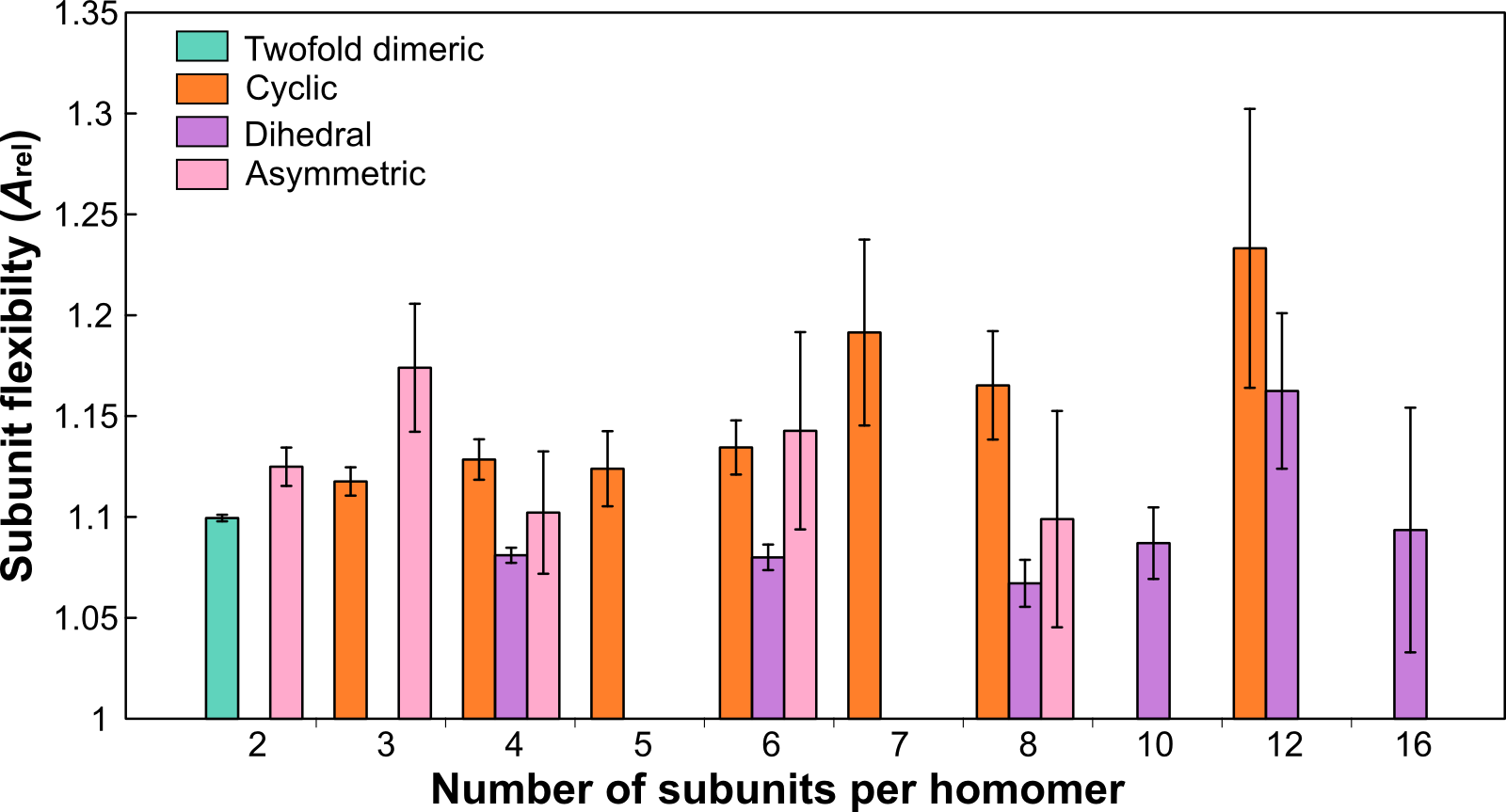

Supplement: Figure S4 — Subunit flexibility is largely independent of the number of subunits in a homomeric complex. Comparison of subunit flexibility, as measured by A rel, to the number of subunits in homomers from different symmetry groups. The overall correlations (r) between A rel and number of subunits are 0.115 for cyclics (p = 0.0002), 0.056 for dihedrals (p = 0.03), and 0.092 for asymmetrics (p = 0.07). Thus, there appears to be a very slight but significant tendency for larger homomers to have more flexible subunits. Error bars represent SEM. (TIFF) [file pbio.1001870.s004.tiff]

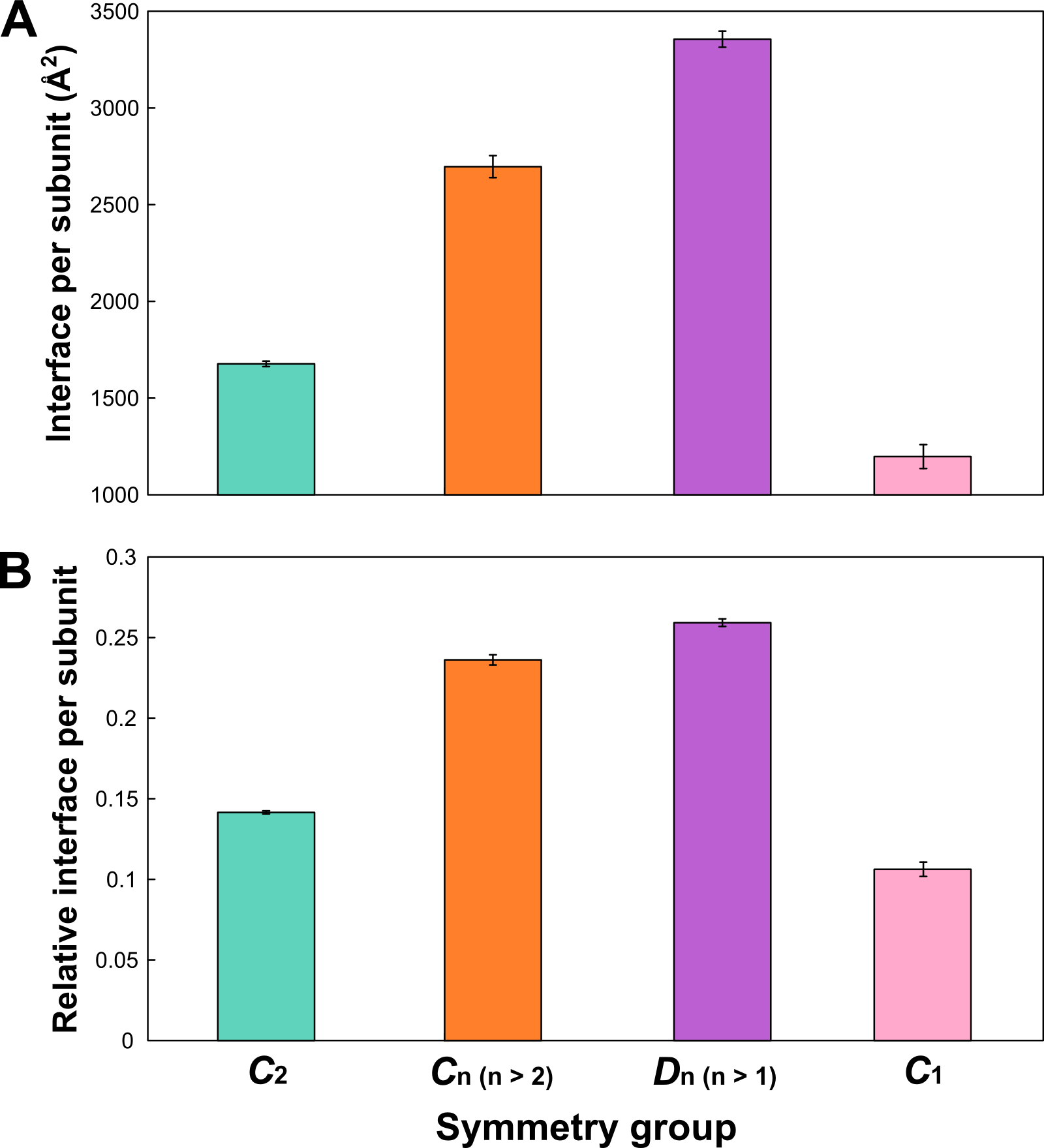

Supplement: Figure S5 — Interface size is related to symmetry but does not explain the observed flexibility trends. Comparison of interface sizes for homomeric subunits in different symmetry groups: (A) mean interface area per subunit; (B) mean relative interface area per subunit (i.e., what fraction of the surface forms interface). Error bars represent SEM. The trends here show essentially no correspondence with the flexibility results in Figure 1A, demonstrating that the association between flexibility and symmetry is not simply due to a requirement to form larger interfaces. (TIFF) [file pbio.1001870.s005.tiff]

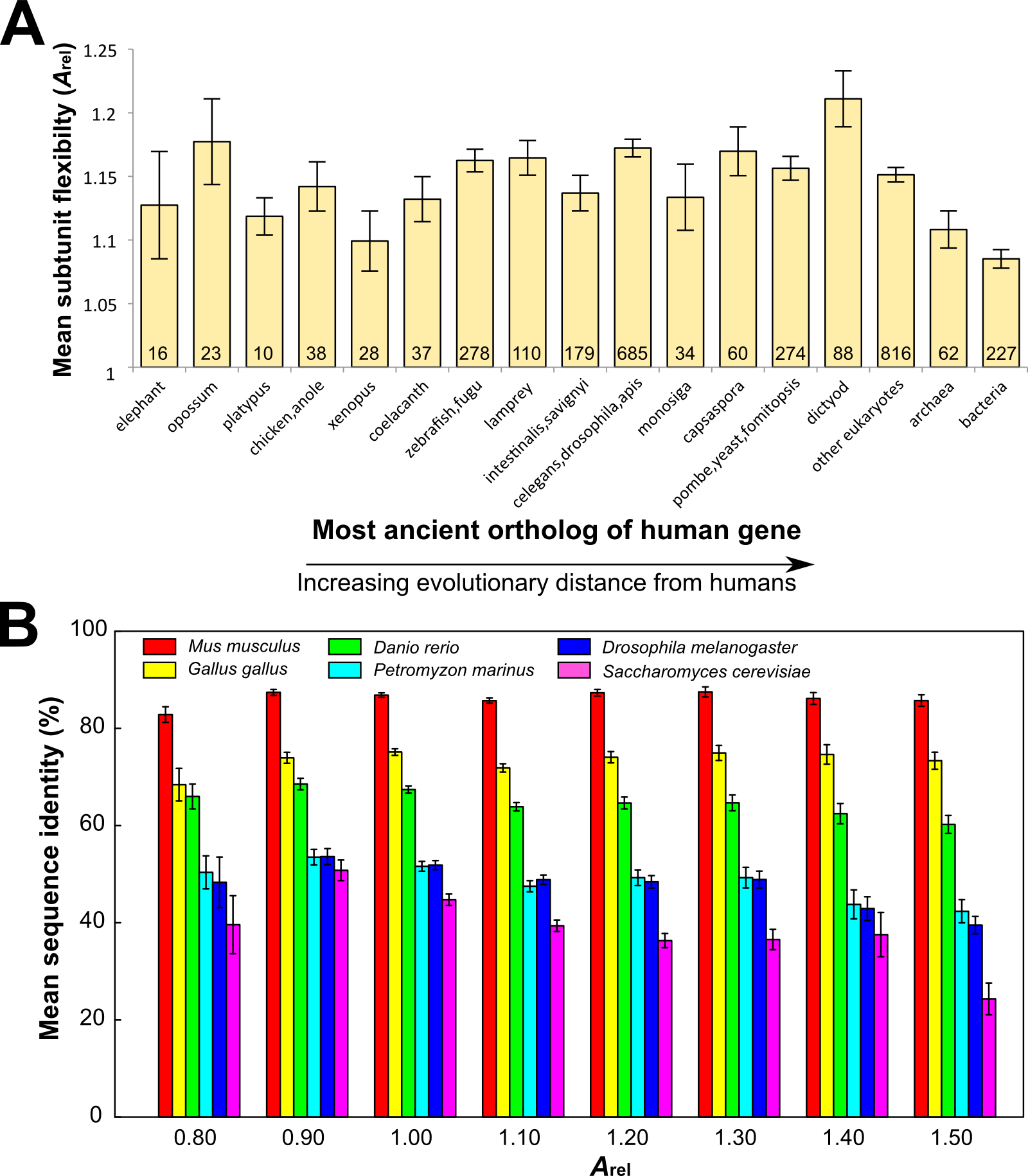

Supplement: Figure S6 — The observation that evolutionarily more recent subunits are more flexible does not arise from a general tendency for increased flexibility in newer proteins. Although we observed a strong trend for the evolutionarily more recent subunits of protein complexes to be more flexible, it is possible that this could to some extent reflect a general tendency for evolutionarily more recent proteins to be more flexible. This could also arise if more flexible proteins tend to evolve at a faster rate, thus making them less likely to be detected as orthologs. We have addressed this in two ways: (A) comparison of A rel values for human (or closely related) subunits whose most ancient orthologs are of varying evolutionary ages. Error bars represent SEM. There is no clear tendency for newer subunits to be more flexible (although subunits conserved in bacteria do appear to be less flexible), suggesting that our results cannot be explained by a general tendency for newer proteins to be more flexible. Full species names and the different evolutionary groups are provided in Table S6. (B) Comparison of sequence identities for subunits of varying flexibility. Here we grouped subunits by A rel and plotted the mean sequence identities of Ensembl Compara orthologs from different species. This shows that, for the most part, sequence conservation is fairly constant with respect to A rel, although there is some tendency for the most flexible human subunits to be less conserved, particularly when compared to yeast. (TIFF) [file pbio.1001870.s006.tiff]

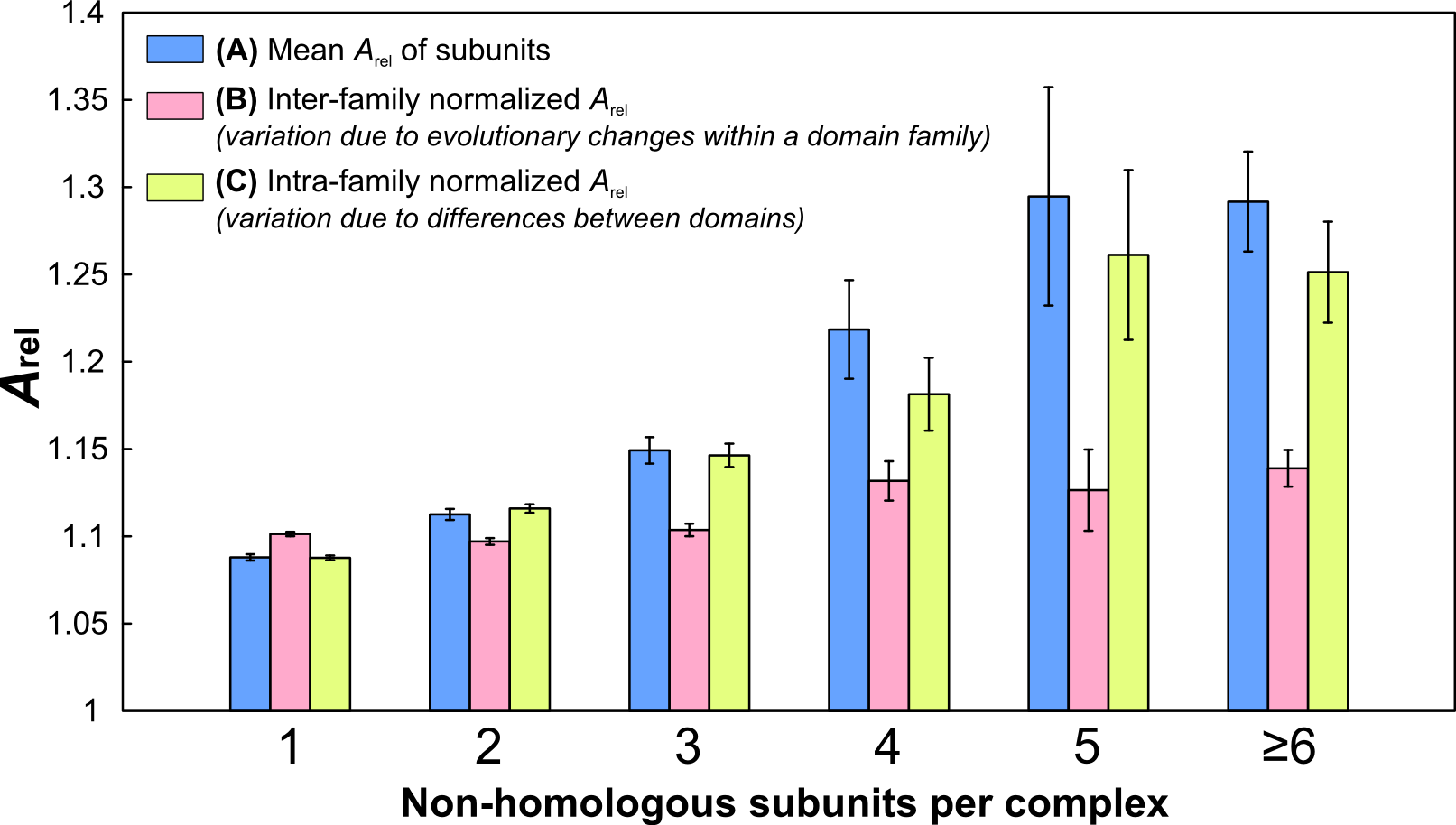

Supplement: Figure S7 — The correspondence between subunit flexibility and the number of nonhomologous subunits per complex is not due to existing subunits evolving to become more flexible. The correspondence between subunit flexibility and the number of nonhomologous subunits per complex could possibly be explained if the existing (i.e., older) subunits of a complex can evolve to become more flexible as new, more rigid subunits are added. To test this, we grouped subunits by their SUPERFAMILY domain architecture. We considered only those groups where evolutionarily related proteins participate in different complexes that have different numbers of nonhomologous subunits. We then plot the relationship between A rel and the number of nonhomologous subunits in three ways (values provided in Table S7): (A) The blue bars are essentially equivalent to Figure 2, although only those subunits that are also considered in (B) and (C) are included here. (B) The pink bars represent the “interfamily normalized” A rel values, in which all variation should be due to evolutionary changes within a domain family. Here, the A rel value for each subunit has been divided by the mean A rel value for all subunits with the same domain architecture. The values are then all scaled by the mean A rel of all subunits in the dataset. If there is a tendency for evolutionarily related proteins to be more flexible when they are part of complexes with more nonhomologous subunits, then we would expect these values to show an increasing trend. However, there is only a very slight trend, which does not explain the variation shown in (A). (C) The yellow bars represent the “intrafamily normalized” A rel values, in which all variation should be due to differences between different types of domains. In these, the A rel value of each subunit has been replaced with the mean A rel value for all subunits with the same domain architecture. Thus we can see that nearly all of the trend in (A) can be explained by differences between evolu [file pbio.1001870.s007.tiff]

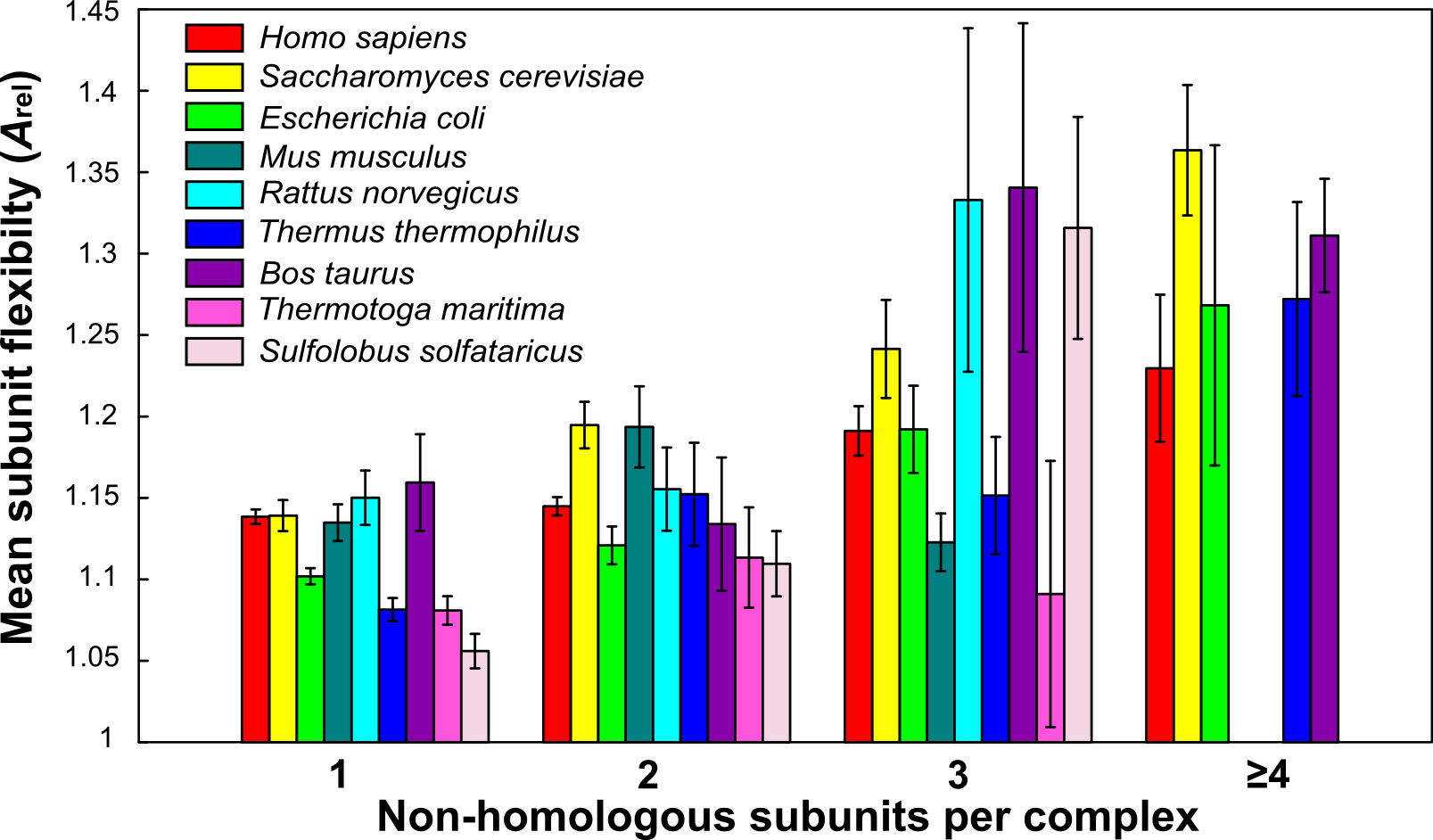

Supplement: Figure S8 — The association between flexibility and the number of nonhomologous subunits per complex is preserved across different species. This plot is essentially the same as Figures 2 and 4C, except it considered separately the nine species with the most heteromers in our nonredundant dataset. A clear trend is observed for nearly all species. Only M. musculus and T. maritima appear to deviate, although this is likely due to the limited size of the dataset, including the fact that no complexes with >3 nonhomologous subunits are present for these species. (TIFF) [file pbio.1001870.s008.tiff]

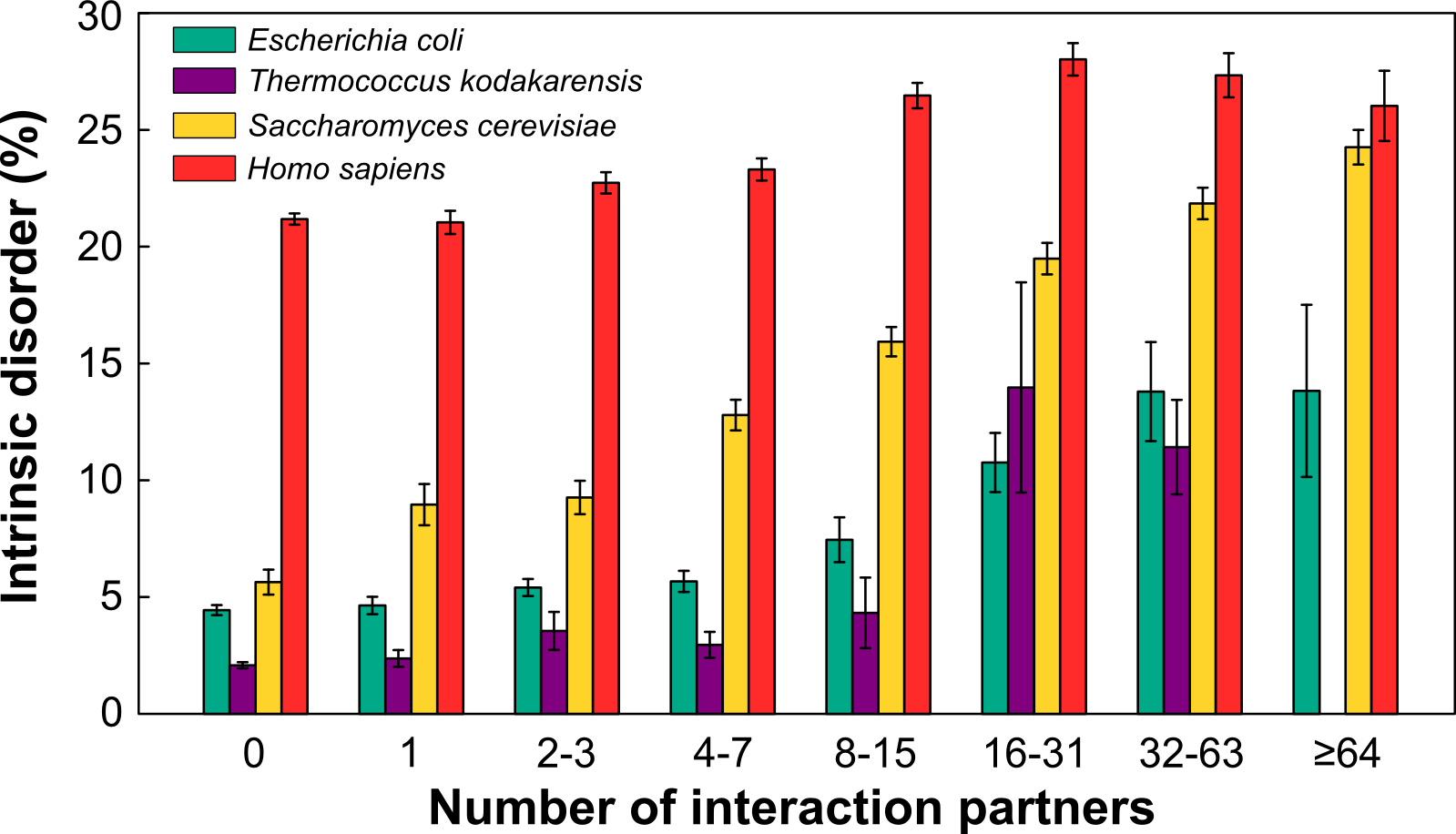

Supplement: Figure S9 — Increasing intrinsic disorder is associated with a greater number of interaction partners across different species. Comparison of the percentage of residues predicted to be intrinsically disordered for proteins grouped by their number of experimentally identified interaction partners. Experimental protein–protein interactions were taken from STRING v9.0 [62], using only interactions with an experimental evidence confidence score >0.3. Varying the threshold from 0.15 to 0.7 preserved the same general trends. The bacterial, archaeal, and two eukaryotic species with the most interactions are shown here. Error bars represent SEM. (TIFF) [file pbio.1001870.s009.tiff]
